# Analysis of the Protective Immunity Induced by Herpes Simplex Virus 1 Strain M3 with an Attenuated Phenotype Due to Mutations in the Viral *ul7*, *ul41*, and *LAT* Genes

**DOI:** 10.3389/fmicb.2017.01958

**Published:** 2017-10-09

**Authors:** Xingli Xu, Shengtao Fan, Xi Wang, Yunguang Hu, Min Feng, Lichun Wang, Ying Zhang, Yun Liao, Xiaolong Zhang, Qihan Li

**Affiliations:** Yunnan Key Laboratory of Vaccine Research and Development on Severe Infectious Diseases, Institute of Medical Biology, Chinese Academy of Medical Sciences and Peking Union Medical College, Kunming, China

**Keywords:** herpes simplex virus 1 (HSV1), protective immunity, M3, ul7, ul41, LAT

## Abstract

Herpes simplex virus 1 (HSV1) is an important pathogen with a worldwide epidemic trend that affects populations of various ages. It has a high morbidity, particularly in juveniles, but a successful HSV1 vaccine is not currently available. Thus, our study systematically observed the immune responses induced in mice immunized with the attenuated HSV1 M3 mutant strain, which has mutations in the genes encoding the UL7 and Vhs tegument proteins and the latency-associated transcript. The immunity induced by the M3 mutant strain can control acute viral infection during HSV1 wild-type strain infection. Moreover, this immunity exerts a potent effect on controlling viral entry into the trigeminal neurons. These data encourage further studies investigating the development of M3 as a potential vaccine candidate, and much work is necessary to evaluate the safety and improve the immunogenicity of this strain.

## Introduction

Herpes simplex virus 1 (HSV1), a member of the herpes virus family, can cause infectious diseases in humans, including herpes labialis, ocular herpes, herpes encephalitis, and genital herpes, which have shown recent increases in morbidity (Xu et al., 2006; Fatahzadeh and Schwartz, 2007; Garland and Steben, 2014). At present, herpes infection exhibits a worldwide epidemic trend. These diseases imperil populations of various ages and cause high morbidity, particularly in juveniles (Smith and Robinson, 2002). HSV1 diseases are characterized by severe pain and discomfort at the herpes lesion sites, resulting in a serious impact on human quality of life (Vyse et al., 2000; Looker et al., 2015). This effect has resulted in public concern regarding the treatment of and prevention measures for these diseases (Michelini et al., 2008; Davola et al., 2015). In parallel, HSV1 studies have provided data that have contributed to our understanding of viral molecular biology and immunology. HSV1 possesses a complicated genomic structure that is associated with its elaborate transcription/replication mechanisms and encodes various viral proteins that function during each stage of infection. The virus presents features of the acute/latent phases of viral infection in nervous tissues, which leads to the failure of specific immunity and therefore the inability to eliminate the entire virus *in vivo* (Egan et al., 2013). Moreover, a successful HSV1 vaccine is not currently available. Many previous works have focused on the development of an HSV1 vaccine (Coleman and Shukla, 2013; Johnston et al., 2016). These studies have provided significant data suggesting that the validity of a vaccine should be dependent on its capacity to ensure a cellular immune response capable of inducing a cytotoxic effect in the vaccinated individual, which is an indicator of the clinical protective efficacy (Bernstein and Stanberry, 1999; Stanfield and Kousoulas, 2015). The attenuated varicella virus vaccine, which is also a member of the herpes virus group, has shown perfect protective effects in its application in children (Sarkadi, 2013), suggesting the possibility of developing an HSV1 vaccine despite the viral preference for nervous system cells during latent infection. Clinical epidemic observations in children who received the varicella vaccine showed not only a dramatic decline in varicella morbidity but also a lower rate of adverse events reported officially over a 10-year period (Johnson et al., 1997; Seward et al., 2002; Wang et al., 2016).

Based on these findings and studies utilizing HSV1 mutants (Read et al., 1993; BenMohamed et al., 2015; Xu et al., 2016), our previous work constructed the M3 HSV1 strain, which contained mutations in the genes encoding the UL7 (ul7) and Vhs (ul41) tegument proteins and the latency-associated transcript (LAT) using the CRISPR/Cas9 method. M3 presented an attenuated phenotype, including a lower rate of viral proliferation in cultured cells, weaker virulence in mice, and lower replication in neurons.

To investigate the immunogenicity of this strain as a vaccine candidate, this study systematically observed the immune response induced in mice immunized with the M3 strain and the clinical protective efficacy provided by this immunity in mice challenged with the wild-type strain. This study also evaluated the safety of M3 in immunized mice by monitoring the viral titration in all tissues, particularly nervous tissues, and the efficiency of viral entry into the trigeminal nerve in mice. All results suggested that this attenuated strain shows potential for further study as an HSV1 vaccine candidate.

## Materials and Methods

### Cells and Viruses

The KMB17 cell line (IMB, CAMS, Yunnan, China) and the African green monkey kidney Vero cell line (ATCC, Manassas, VA, United States) were maintained in high glucose Dulbecco’s modified Eagle’s medium (Corning, NY, United States) supplemented with 10% fetal bovine serum (HyClone, Logan, UT, United States). The culture medium was changed to DMEM supplemented with 2% fetal bovine serum after viral infection. The pathogenic HSV-1 strain 8F (Yu et al., 2010) and the HSV1 mutant M3 were used in our experiments. The mutant M3 was originated from HSV-1 strain 8F, in which a 30-bp (225–254) sequence of the ul7 gene, a 59-bp (375–433) sequence of the ul41 gene, and a 138-bp (937–1074) sequence of the LAT gene were deleted sequentially using the CRISPR/Cas9 method. The mutants were identified by PCR and sequencing of the PCR products, and the mutated clones were acquired through plaque screening of the harvest in Vero cells. The viruses were titered on Vero cells.

### Mouse Study Design and Ethics Statement

Four-week-old female BALB/c mice weighing 10–13 g (Vital River, Beijing, China) were purchased and housed in a specific pathogen-free facility of the Institute of Medical Biology. The mice were maintained under a 12-h light/dark cycle (lights on at 08:00 h) at 22 ± 1°C. The animals were housed individually and allowed free access to food and water. All efforts were made to minimize suffering or discomfort to the animals. Prior to the experiments, the animals were routinely acclimated (>1 week) to laboratory conditions to reduce potential stress effects during the experiments. The animal experiments were designed based upon the principles expressed in the “Guide for the Care and Use of Laboratory Animals” and “Guidance for Experimental Animal Welfare and Ethical Treatment.” The experimental protocols were reviewed and approved by the Yunnan Provincial Experimental Animal Management Association (approval number: SCXK [Dian] 2011-0005) and the Experimental Animal Ethics Committee of the Institute (Approval number: YIKESHENGLUNZI [2016] 54).

For the immunization experiment, the BALB/c mice were narcotized under 2% isoflurane inhalation and immunized via an intramuscular injection of 10^3^, 10^4^ or 10^5^ plaque-forming units (PFUs) of the HSV-1 mutant M3 or phosphate-buffered saline (PBS, sterile, pH 7.4) as a control. Splenic lymphocytes were isolated 1 and 2 months post-immunization for an ELISpot assay. Additionally, blood samples from the mice were tested for neutralizing antibodies on days 28, 42, and 56 after the primary injection.

After determining the optimal immunization dose, the BALB/c mice were immunized via an intramuscular injection of 10^4^ (PFUs) of the HSV-1 mutant M3 or PBS (sterile, pH 7.4) as a control. At 1 and 2 months post-immunization, the immunized and PBS-immunized mice were challenged with HSV1 8F, McKrae, and 17+ (2 × 10^4^ PFU/50 μL/mouse) via the respiratory tract. After the viral challenge, the weights of the mice were measured every 2 days. The survival rate was assessed over a 10-day period. The splenic lymphocytes were isolated 6 days post-viral challenge for an ELISpot assay. Tissues were obtained at 3, 6, and 9 days post-viral challenge and subjected to an assessment of mouse organ pathology.

### Virus Titration

Virus titration was analyzed by performing a micro-titration assay according to a standard protocol. For the titration of various mouse organs, viral stocks were ground and homogenized as much as possible, and the homogenate supernatants were then serially diluted 10 times and added to 96-well plates coated with Vero cells. The plates were incubated at 37°C in 5% CO_2_ and scored for the presence of cytopathic effect (CPE) 7 days post-infection. All virus-related experiments were performed in a large room under BSL-2 conditions.

### Neutralization Assay

A neutralization assay was performed in accordance with standard protocols. Briefly, a mixture of diluted serum (1:4, 1:8, 1:16, 1:32, and 1:64) and virus at a titer of 100 times the 50% cell culture infectious doses (CCID_50_)/100 μL were incubated at 37°C for 2 h. The mixture was then added to Vero cells in 96-well plates and incubated at 37°C. The CPE of the virus was observed after 1 week.

### Preparation of Mouse Splenic Lymphocytes for the Experiments

BALB/c mice were sacrificed after anesthetization with ether, and the spleens were removed aseptically into Hank’s Balanced Salt Solution (Corning, NY, United States). A single-cell suspension was prepared through the gentle dispersion of the cells. Red blood cells were removed using 5 mL of mouse lymphocyte separation solution (Solarbio, Beijing, China) per mouse spleen for 30 min. The cells were washed and suspended in RPMI 1640 medium (Corning, NY, United States) supplemented with 10% heat-inactivated fetal calf serum (FCS), 100 U/mL penicillin, and 100 μg/mL streptomycin.

### IFN-γ-Specific ELISpot Assay

Splenic lymphocytes were isolated as described above. A mouse IFN-γ ELISpot Kit (MABTECH Inc., Cincinnati, OH, United States) was used according to the manufacturer’s protocol. Briefly, the plate was conditioned and seeded with splenic lymphocytes prior to addition of the stimulant (the two 95% purity peptides: gB498-505: SSIEFARL and ICP6822-829: QTFDFGRL), which mainly targeted the CD8^+^ T lymphocytes (Salvucci et al., 1995; Wallace et al., 1999; St Leger et al., 2011). The cells were then incubated at 37°C for 24 h. After incubation, the cells were removed, and the plate was developed. The colored spots were counted using an automated ELISpot reader (CTL, Cleveland, OH, United States), with spot-forming cells (SFCs) representing HSV1 M3-specific IFN-γ-producing T cells.

### Histopathological and Immunohistochemical Examinations

The mouse organs were fixed in 10% formalin and embedded in paraffin into tissue blocks. Approximately two slides per organ were stained with hematoxylin and eosin (H&E) to assess the morphology. For the immunohistochemical examinations, the specimens were immersed in 0.3% hydrogen peroxide in PBS for 10 min to block the intrinsic peroxidase activity. After rinsing with distilled water, the specimens were heated at 90°C for 30 min for antigen retrieval. The specimens were blocked with 5% bovine serum albumin (BSA) in PBS at 37°C for 15 min. The specimens were then incubated with a rabbit polyclonal anti-HSV-1 antibody (Abcam, Cambridge, United Kingdom) at 4°C for 12 h. The sections were incubated with poly-HRP anti-rabbit IgG (ZSGB-BIO, Beijing, China) at 37°C for 30 min after rinsing with PBS. Enzyme immunohistochemistry was performed using a standard avidin-biotin-peroxidase method with the 3,3′-diaminobenzidine (DAB) substrate (Tiangen, Beijing, China) according to the manufacturer’s recommended protocol. After rinsing with water for 6 min, the sections were stained with hematoxylin to assess the cell nuclei. For each staining condition, a slide with rabbit IgG (Abcam, Cambridge, United Kingdom) was stained and examined in parallel as a control.

### *In Situ* Hybridization

An *in situ* hybridization assay was performed using the Enhanced Sensitive ISH Detection Kit I (POD) (Boster, Wuhan, China) per the manufacturer’s recommendations. Briefly, frozen sections were fixed with 4% paraformaldehyde for 20 min at room temperature. The sections were immersed in 0.6% hydrogen peroxide in methanol to block the intrinsic peroxidase activity. After three rinses with distilled water, pepsin digestion was performed, followed by treatment in pre-hybrid solution for 3–4 h. Twenty microliters of the LAT probe (5′-CATAGAGAGCCAGGCACAAAAACAC-Dig-3′, 1 μg/mL hybrid solution) was pipetted onto each sample and left overnight in a humidity chamber. The sections were consecutively treated with sealing liquid for 30 min, mouse-anti-DIG for 60 min, SABC solution for 20 min, anti-mouse-HRP polymer for 20 min, and diaminobenzidine chromogen (DAB) for 10 min. After rinsing with water, the sections were stained with hematoxylin to assess the cell nuclei. LAT positivity was evaluated in the entire cell. Any brown dot-like signal from the cell was considered positive for LAT expression. Cells with no such signal were considered negative for LAT.

### Co-culture of Trigeminal Ganglion Tissues and Vero Cells

Trigeminal ganglions were removed from the sacrificed mice 9 days post wild-type virus challenge. The tissues were cut into smaller sections and placed on monolayers of Vero cells. CPE was monitored daily for 7 days. If no CPE was detected, the cultures were continuously blind passaged for three generations and the virus titrations of the third generation were determined by the standard protocol on Vero cells. Meanwhile, DNA was extracted from the culture mediums for PCR testing of HSV1. The genomic regions of the RS1, ul48, and ul44 genes were amplified by PCR using specific primers. The specific primer sets used are listed in **Table 1**.

**Table 1:** Primers used for PCR.

Primer name	Assay	Genomic target	Primer sequence (5′ to 3′)
RS1-F	PCR	RS1	CTGCTGGCCTCCATGGTAGA
RS1-R			TCATCGTCGTCGGCTCGAA
Ul48-F	PCR	Ul48	CAGCGATGTGGTGGAATG
Ul48-R			CCAACACGGTTCGATAGC
Ul44-F	PCR	Ul44	CGGATGGCACGAGTTCAA
Ul44-R			AGGATCTTGCCGACTGGA

### Statistical Analysis

The results of the various assays, which were performed in triplicate, are expressed as mean values with standard deviations. SPSS software was used for statistical analyses. The weights of the infected mice were evaluated using repeated measures. A survival analysis was performed to analyze the survival rate of infected mice. Differences of virus titers between two groups were evaluated using an independent sample *t*-test. A *p*-value of *p* < 0.05 was considered significant.

## Results

### Intramuscular Inoculation of the HSV1 M3 Mutant Induces an Immune Response in Mice

**FIGURE 1:**
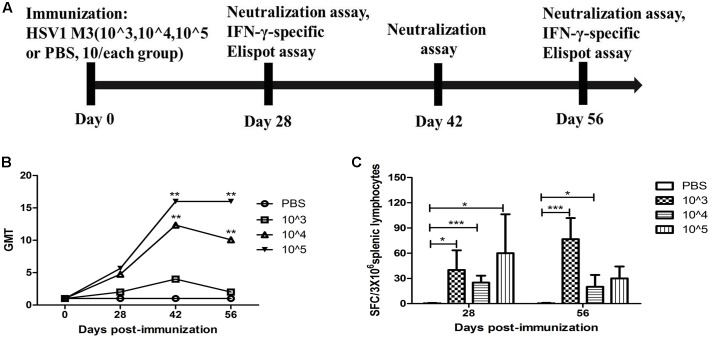
Immune response to herpes simplex virus 1 (HSV1) induced in mice immunized with the HSV1 mutant M3. **(A)** Design of the immunological analysis for the evaluation of M3. **(B)** Neutralizing capability of the antibodies against HSV1 8F in mice immunized with HSV1 M3 (*n* = 5 mice per dose) or phosphate-buffered saline (PBS) (*n* = 5). The geometric mean titers (GMTs) of the neutralization antibodies were measured by a neutralization test as described in the “Materials and Methods.” The GMT values for the negative control (PBS) groups were all <2. **(C)** The ELISpot responses to IFN-γ-secreting cells from splenic lymphocytes in the HSV1 M3-immunized (*n* = 5 mice per dose) and control mice (PBS, *n* = 5). Splenic lymphocytes were incubated for 24 h in the presence of the stimulator. The positive control (Pos. con) was phytohemagglutinin (PHA). The values are presented as the means ± SD. ^∗^*p* < 0.05; ^∗∗∗^*p* < 0.001.

**FIGURE 2:**
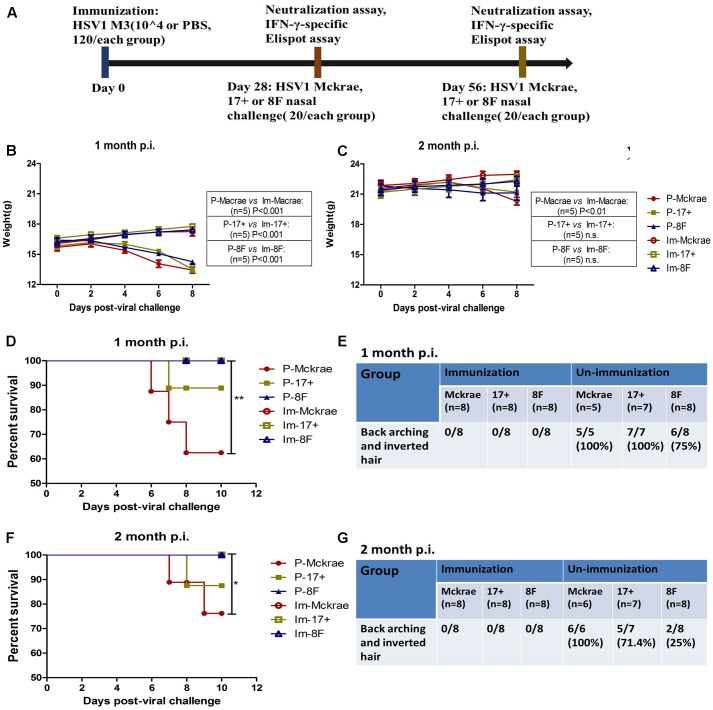
Clinical observations of the immunized and unimmunized mice after viral challenge. **(A)** Design of the mice experiment for the wild-type (WT) virus challenge. Mice immunized with M3 were challenged with the HSV1 WT strains McKrae (Im-McKrae, open circles), 17+ (Im-17+, open squares), and 8F (Im-8F, open triangles) via the intranasal instillation of 2 × 10^4^ plaque-forming unit (PFU). Mice immunized with PBS were also challenged with the HSV1 WT strains McKrae (P-McKrae, filled circles), 17+ (P-17+, filled squares), and 8F (P-8F, filled triangles). **(B,C)** The weights of the mice were measured every 2 days. **(D–G)** The morbidities and survival rate of the mice infected with the virus were observed after a 10-day period. The data are shown as the means ± SD. ^∗^*p* < 0.05; ^∗∗^*p* < 0.01; ^∗∗∗^*p* < 0.001.

Based on the observation that M3 had an attenuated phenotype in mice, our experiments utilized high, medium and low M3 doses (10^5^, 10^4^, and 10^3^ PFU/mouse) for immunization via intramuscular injection into the mice. Each dose group contained 10 mice, which were used for immunological detection at two time points (1 and 2 months post-immunization; **Figure 1A**). All mice inoculated with any dose of the mutant strain developed a specific neutralizing antibody response in a dose-dependent manner (**Figure 1B**). An ELISpot response against the HSV1 gB and ICP6 antigens was observed in T cells that specifically secreted IFN-γ (**Figure 1C**). These immunological indicators were up-regulated in the immunized mice, indicating that M3 can induce specific immunity against HSV1. Also, in our previous work, we evaluated the immune protective efficiency of three doses. The results showed that 10^3^ PFU/mouse was ineffective to induce favorable immune response, whereas the mice immunized with 10^4^ PFU/mouse and 10^5^ PFU/mouse doses showed a similar immune protective efficiency (data not shown). Therefore, we prefer a lower dose of 10^4^ PFU/mouse to obtain the same immune protective effect with a higher safety. Hereafter, 120 mice were immunized with M3 (with a dose of 10^4^ PFU/mouse) via intramuscular injection, and 60 were used for the viral challenge at each time point. In parallel, an equal number of mice were challenged with the wild-type strains 8F, McKrae, and 17+ at the same dose as positive controls (**Figure 2A**). The immune response, including neutralizing antibodies and a specific ELISpot response against gB and ICP6 antigen peptides, was assessed 1 and 2 months after one injection of this virus followed by challenge with the HSV1 wild-type strains 8F, McKrae, and 17+ (2 × 10^4^ PFU/mouse) via the nasal mucosa (**Figure 2A**).

### Comparative Clinical Observations of M3-Immunized and Unimmunized Mice during Challenge with the HSV1 Wild-Type Strain

Clinical observations of the mice immunized with M3 did not reveal any visible symptoms, which suggested an attenuated phenotype of M3, similar to the findings of our previous work. Interestingly, we did not find any clinical changes in the immunized mice at 1 or 2 months after challenge with the three wild-type strains. The weight measurements of all the mice indicated that the immunized mice maintained their increasing weight trend over 10 days post-infection. In contrast, a decreasing trend was observed in the unimmunized mice over the same period (**Figures 2B,C**). Moreover, all of the animals in the immunized and 8F challenged (P-8F) groups survived at 1 month post-immunization, whereas three of eight mice (37.5%) in the McKrae challenge (P-McKrae) group and one of eight mice (12.5%) in the 17+ challenge (P-17+) group died (**Figure 2D**). All animals in the P-McKrae and P-17+ groups became ill, with most of the mice exhibiting symptoms of severe disease, including back arching and inverted hair, whereas six of the eight animals in the P-8F group developed back arching and inverted hair, and no animals in the immunization groups displayed these complications, at 1 month post-immunization (**Figure 2E**). In addition, at 2 months post-immunization, two of eight mice (25%) in the McKrae challenge (P-McKrae) group and one of eight mice (12.5%) in the 17+ challenge (P-17+) group died, whereas all of the animals in the other immunization groups survived (**Figure 2F**). All animals in the P-McKrae developed back arching and inverted hair, whereas five of seven animals in the P-17+ group and two of eight animals in the P-8F group developed back arching and inverted hair, and no animals in the immunization groups displayed these complications (**Figure 2G**).

### The Pathological Manifestations in Immunized and Unimmunized Mice Post-Viral Challenge

**FIGURE 3:**
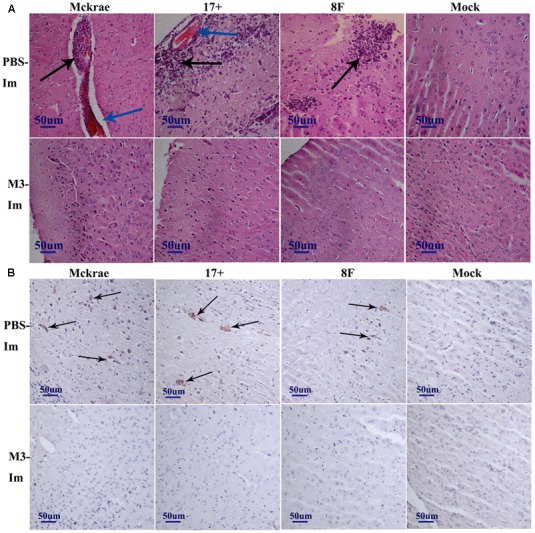
The pathological and immunohistochemical analyses of the immunized and unimmunized mice after viral challenge. **(A)** Pathological changes in cerebral tissues of mice challenged with the HSV1 WT virus (3 days post-viral challenge, 3 d.p.i., or 6 days post-viral challenge, 6 d.p.i.) at 1 or 2 months post-immunization. The tissue sections were stained with hematoxylin and eosin (H&E) and imaged using an optical microscope at 200× magnification. Tissue hyperemia is highlighted with a blue arrow, and the infiltration of inflammatory cells are highlighted with a black arrow. The mice challenged with PBS were the negative control. **(B)** HSV-1 immunohistochemistry of the cerebral tissues of mice challenged with HSV1 WT (3 days post-viral challenge, 3 d.p.i., or 6 days post-viral challenge, 6 d.p.i.) at 1 or 2 months post-immunization. Positive expression of the HSV-1 antigen was detected in the PBS-immunized mouse cerebral tissues (arrows). Mice challenged with PBS served as the negative control.

The pathological assessment of the major organs of all mice post-viral challenge was an important evaluation of the immunological protective effect. The assessment suggested a trend in which the pathological changes showed the presence of fewer inflammatory lesions in the mice immunized with the M3 strain than in the unimmunized mice (**Table 2**). The observations focused on the cerebrum indicated an obvious inflammatory lesion and infiltration of inflammatory cells in the cerebrum in the unimmunized animals challenged with all three wild viral strains since day 3 post-viral challenges (**Figure 3A**). These lesions appeared to extend to more areas with more inflammatory cell aggregation around vesicular tissues, whereas the degeneration of some neurons associated with congestion in the tissues were presented on day 6 (**Figure 3A**). Moreover, inflammatory cell infiltration and bleeding in the meninx were observed in most of the mice. Conversely, the mice immunized with the M3 strain showed slight inflammatory cell aggregation and a few areas presenting slight congestion in the local area of the meninx (**Figure 3A**). These pathological changes presented a distinct trend at various time points in the immunized and unimmunized mice. We performed an immunohistochemical detection using a specific antibody against HSV1 on the same sections obtained from all mice. In cerebral sections from the unimmunized animals, many dispersed HSV1 antigenic-positive signals were observed in the areas with aggregated inflammatory cells (**Figure 3B**). In contrast, few antigen-positive signals were observed in the local tissues of the cerebrum, where inflammatory cells aggregated in the immunized mice (**Figure 3B**). These results indicated that the immunity induced in the mice immunized with M3 might be capable of controlling the pathological lesions induced by infection with the HSV1 wild-type strain.

**Table 2:** Neurovirulence of M3-immunized and unimmunized mice challenged with 2 × 10^4^ plaque-forming unit (PFU)/50 μL of wild-type herpes simplex virus 1 (HSV-1).

Days post-viral challenge	Groups (cerebrum)											

	P-McKrae		P-17+		P-8F		Im-McKrae		Im-17+		Im-8F	
	1 m.p.i.	2 m.p.i.	1 m.p.i.	2 m.p.i.	1 m.p.i.	2 m.p.i.	1 m.p.i.	2 m.p.i.	1 m.p.i.	2 m.p.i.	1 m.p.i.	2 m.p.i.
3	+	+	+	+	+	+	±	±	±	±	±	±
6	++	+	++	+	++	+	±	±	±	±	±	±
9	++	+	++	+	+	+	±	±	±	±	±	±

*Scale as follows: Grade -, normal tissue, Grade +, slight infiltration of inflammatory cells without neural damage, Grade ++, slight neural damage with inflammatory cell infiltration, Grade +++, massive neural damage with inflammatory cell infiltration, Grade ++++, serious neural damage with inflammatory cell infiltration.*

### The Immunity Induced by M3 Can Inhibit Viral Replication in Mice

**FIGURE 4:**
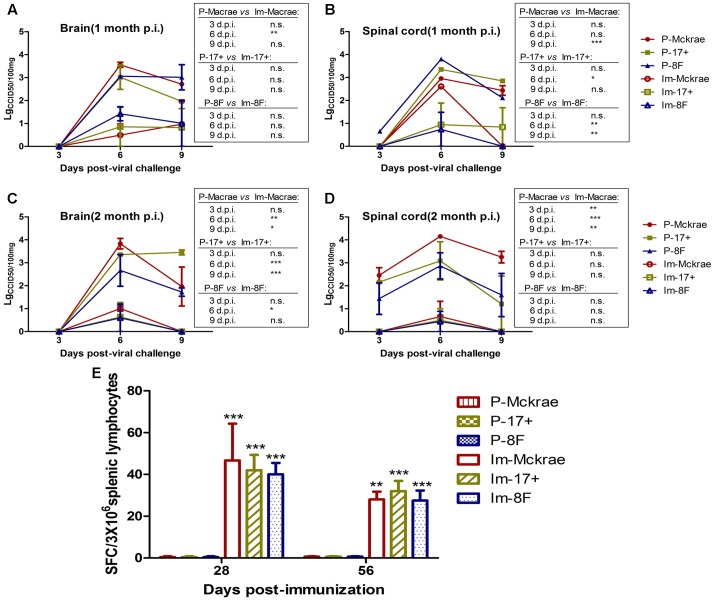
The immunity induced by M3 can inhibit viral replication in immunized individuals. **(A,B)** Viral titration detection in the brain **(A)** or spinal cord **(B)** of mice immunized with M3 (or PBS) and challenged with the HSV1 WT strains McKrae, 17+ or 8F at 1 month post-immunization. **(C,D)** Viral titration detection in the brain **(C)** or spinal cord **(D)** of mice immunized with M3 (or PBS) and challenged with the HSV1 WT strain McKrae, 17+ or 8F at 2 months post-immunization. **(E)** The ELISpot responses to IFN-γ-secreting cells from splenic lymphocytes in the HSV1 M3-immunized mice (*n* = 3 mice per dose) and the mock mice (PBS, *n* = 3) at day 6 post-viral challenge. The splenic lymphocytes were incubated for 24 h in the presence of the stimulator. The positive control is PHA. The data are shown as the means ± SD. ^∗^*p* < 0.05; ^∗∗^*p* < 0.01; ^∗∗∗^*p* < 0.001.

The above comparative observations of the pathological changes induced by infection with the three wild viral strains in the immunized and unimmunized mice suggested that the severity of the pathological lesions was controlled in the immunized mice. To verify this observation, we evaluated the dynamic variations in the viral titration in the major organs of the immunized and unimmunized mice at 3, 6, and 9 days post-viral challenge. The viral titration for the CNS tissues, including various parts of the brain and spinal cord from the immunized mice, suggested a lower trend than those detected in the organs from the unimmunized mice (**Figures 4A–D**). However, no virus was traced in the other organs, not only in the immunized mice but also in the unimmunized mice. These data suggest that the immunity induced by M3, which can limit the pathological damage of nervous tissues and other tissues caused by the wild viral strain challenges, might be due to the inhibition of the proliferation of challenged virus in immunized individuals. Previous studies suggested that specific CD8+ cells play an important role in controlling herpes virus infection via a cytotoxic effect against infected host cells (Macleod et al., 2014). Therefore, our work examined specific T cell responses against the viral gB and ICP6 antigens using an ELISpot assay to assess IFN-γ specificity in all mice on day 6 post-viral challenge. We observed an increased specific ELISpot response in the immunized mice relative to that in the unimmunized mice (**Figure 4E**). This finding supports our prediction discussed above.

### The Immunity Induced by M3 Can Control the Entry of the Challenge Strains to the Trigeminal Nerve in the Acute Infectious Period

**FIGURE 5:**
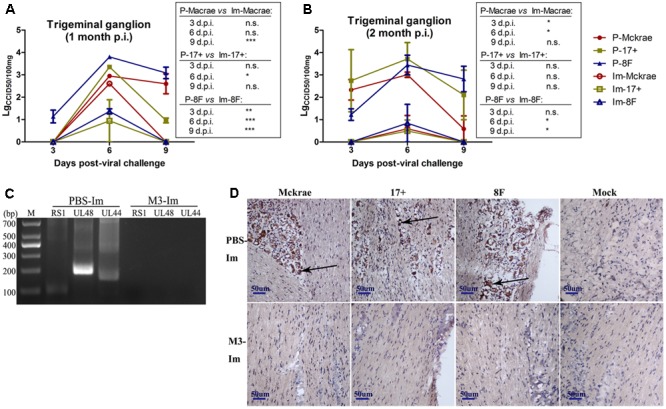
The immunity raised in the immunized mice by M3 can reduce the infectivity of the HSV1 WT strain. **(A,B)** Viral titration detection in the trigeminal ganglion of the mice immunized with M3 (or PBS) and challenged with the HSV1 WT strains McKrae, 17+ or 8F on various days post-viral challenge. The mice were challenged with the HSV1 WT virus via an intranasal instillation of 2 × 10^4^ PFUs at 1 month **(A)** or 2 months **(B)** post-immunization. **(C)** Viral genome detection in trigeminal ganglion tissues by PCR amplification of DNA with primers targeting the RS1 (101 bp), ul48 (191 bp) and ul44 (158 bp) genes. The PCR fragments of three strains (McKrae, 17+ and 8F) were similar in length. **(D)** Positive LAT RNA expression in the trigeminal nerves in mice detected by chromogenic *in situ* hybridization is highlighted with black arrows. Brown-dot positivity was observed in the PBS-immunized mice. A dispensable positive signal was observed in the M3-immunized mice (*in situ* hybridization; 200x). ^∗^*p* < 0.05; ^∗∗^*p* < 0.01; ^∗∗∗^*p* < 0.001.

**FIGURE 6:**
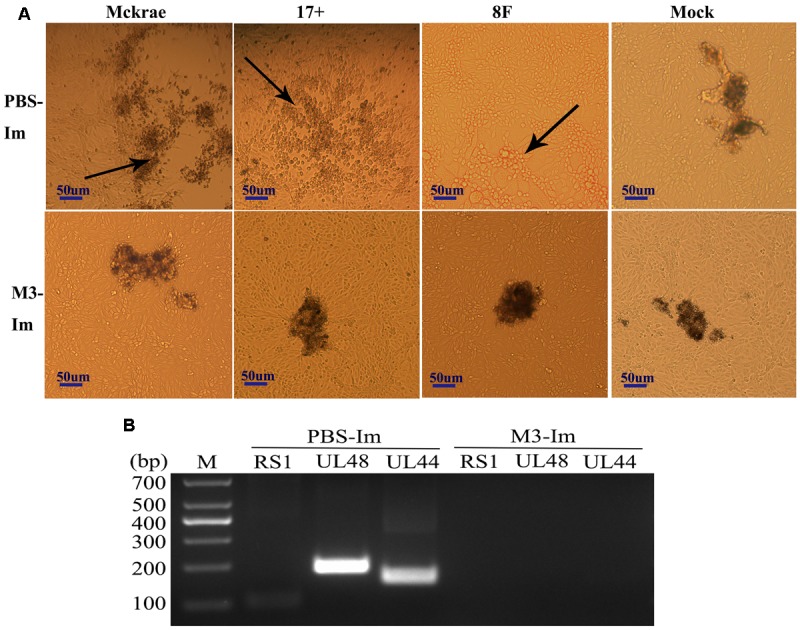
The latent infection of the HSV1 WT is reduced in the mice immunized with M3. **(A)** Co-cultured Vero cells with trigeminal ganglion of immunized and unimmunized mice at 6 or 9 days post-viral challenge. CPE is indicated by black arrows; scale bars = 50 μm. **(B)** Viral genome detection of a mixture of trigeminal ganglion tissues and co-cultured Vero cells by PCR amplification of DNA with primers targeting the RS1 (101 bp), ul48 (191 bp), and ul44 (158 bp) genes. The PCR fragments of three strains (McKrae, 17+, and 8F) were similar in length.

Reported studies of HSV1 infection in mice indicated that the virus could enter the trigeminal ganglion of mice for further possible latent infection while it induced acute infection in CNS tissues (Curanovic and Enquist, 2009). However, some works also suggested no positive data supporting the autonomic reactivation of latent HSV1 in the trigeminal ganglion of mice, even when the expression of LAT RNA was traced in this tissue (Webre et al., 2012). In this study, after 1 and 2 months of immunization with the M3 strain, the mice were challenged with three wild strains: McKrae, 17^+^, and 8F. During the observation period for acute infection, the trigeminal nerve samples obtained from all mice at days 3, 6, and 9 post-viral challenges were used for titration of the virus in this tissue followed by the identification of viral genomic DNA using PCR reaction; simultaneously, an *in situ* hybridization assay was performed to probe the LAT RNA expression to verify the viral transcription in the neurons. The results indicated significantly lower viral loads in the trigeminal ganglions of the mice immunized with the M3 strain than that in the unimmunized mice (**Figures 5A,B**). These results suggest that the immunization of M3 provided a mechanism to control the virus entering the trigeminal neurons. The identification using PCR with primers against the RS1, ul48, and ul44 genes of HSV1 was supportive to this suggestion (**Figure 5C**). As further evidence, the *in situ* hybridization detection showed a significantly lower LAT RNA level in the trigeminal nerves of the immunized mice than that in the unimmunized mice at days 6 and 9 (**Figure 5D**). Subsequently, homogenized trigeminal tissues were co-cultured with Vero cells to investigate the viral reactivation to obtain further evidence to support this immunity. The results indicated that the Vero cells co-cultured with trigeminal ganglion tissue from the control mice challenged by a wild-type strain, particularly P-17+, showed typical CPE (**Figure 6A**, Upper); and no CPE was observed in the cells in immunized mice after viral challenge, even after three passages (**Figure 6A**, Bottom). In addition, PCR using primers targeting the RS1, ul48, and ul44 genes of HSV1 supported these results (**Figure 6B**). These data support the conclusion that M3 immunization in mice establishes an effective clinical immune protection capable of defeating various wild strain challenges.

## Discussion

In the present study, we systematically observed the immune responses induced in mice immunized with the attenuated HSV1 M3 mutant strain, which has mutations in the genes encoding the UL7, Vhs tegument proteins, and the LAT. Firstly, this study focused on the protective efficacy induced in mice immunized with M3 followed by challenge with three wild-type HSV1 strains. Our data suggested that immunity characterized by a neutralizing antibody response and an active cellular immune response was capable of restricting various pathological changes and the associated clinical symptoms induced by viral infection. In contrast to the unimmunized mice, the immunized mice treated with the M3 strain did not show any symptoms of viral infection, such as weight loss, poor physical condition, an arched back, inverted hair, and death in the mock controls. Moreover, the immunized mice maintained 100% health status until sacrifice. This immunological protective effect was supported by the clinical manifestations and pathological and virus titer analyses of tissues, which suggested that restricted viral replication *in vivo* led to reduced tissue lesions. This process might be mediated by the up-regulation of specific CD8**^+^** T cell cytotoxicity against virus-infected cells in the immunized mice after stimulation by viral challenge, which was manifested as an increased T cell response and the secretion of IFN-γ against specific HSV1 antigens in the ELISpot assay. This result indicates that the immunological defense mechanism can control acute viral infection associated with pathological damage, particularly in nervous tissues, during HSV1 wild-type strains infection.

Second, clinical observations of the mice immunized with M3 did not reveal any visible symptoms, suggesting an attenuated phenotype of M3. In addition, fewer viruses were detected in the nervous system, and no virus was detected in other organs after wild-type viral challenge. Furthermore, we investigated the virus titer and virus reactivation in the trigeminal nerves of the mice after 9 days post-viral challenge. The results suggested that the immunity induced by the mutant M3 had a potent effect on limiting viral entering into the trigeminal nerve and there was no virus reactivation in immunized mice after viral challenge. The infection pathology and immunogenicity results revealed that mutant M3 may be a favorable attenuated strain and become a live-attenuated vaccine candidate.

Most studies investigating prophylactic vaccines for HSV1 have evaluated viral structural proteins with antigenicity for safety reasons due to the potent risk of the virus presenting latency and reactivation in nervous tissues, particularly in the trigeminal nerve (Khan et al., 2015; Srivastava et al., 2015). However, few of these vaccine candidates have provided satisfactory data supporting their clinical protective efficacy in human trials to date. These results might be due to the complicated interaction of viral encoding molecules and the immune system, which could lead to an integrated immunity characterized by both specific CD8**^+^** cytotoxicity and a neutralizing antibody response (Staats et al., 1991; Neumann et al., 2003). Amazingly, many works revealed HSV1 strains can be artificially attenuated using a genomic modification method (Forrester et al., 1992; Iyer et al., 2013). In addition, the successful application of an attenuated vaccine for varicella, a member of the herpes virus family, can decrease the morbidity of varicella infection in children for more than 10 years (Johnson et al., 1997). Moreover, few data have been reported for the establishment of latent viral infections and viral reactivation in neurons in vaccinated individuals (Wang et al., 2016). This finding provides the possibility that a viral strain with reduced neurogenic virulence created by an artificial method might be a significant means to induce effective immunity against HSV infections in humans under restricted verification conditions (Morrison and Knipe, 1997). Based on the copious data generated by HSV1 genomic structural and functional studies, the genes involved in the virulence and efficiency of viral entry into trigeminal ganglion neurons have been confirmed in multiple studies though artificial modification targeting. These works include investigations of attenuated and gene-deficient strains (Nguyen et al., 1992; Mao and Rosenthal, 2003; David et al., 2008; Matundan et al., 2015). The mutant M3 used in this work was designed based upon an understanding of the biological characteristics of UL7, Vhs, and LAT during viral infection. In particular, the UL7 tegument protein may be a factor restricting viral proliferation (Xu et al., 2016); the Vhs protein is capable of preventing antigen signal transfer to adaptive immunity from the innate immune response during infection (Samady et al., 2003; Paludan et al., 2011); and the *LAT* gene is related to latency establishment and maintenance (Thompson and Sawtell, 1997). In the current study, specific modifications regarding these proteins create a strain with lower virulence and a reduced latent infection capacity *in vivo*. Our results offer the feasibility of this approach for the construction of a live-attenuated vaccine candidate.

Taken together, our study indicated the favorable immune protective efficiency of M3 strain in mice model. However, in consideration of the complicated immune response of HSV1 infection in human, further modification in the viral proteins related to immune regulation may be needed. For example, ICP47 has been shown to downregulate MHC-I expression and thereby decreasing immune recognition by cytotoxic T-lymphocytes; US3 is involved in modulating the expression of the key antigen presenting molecules CD1d to evade the antiviral function of natural killer T cells; and UL24 is found to inhibit DNA sensor-mediated IFN-β and IL-6 production (Cioni et al., 2013; Xiong et al., 2015; Xu et al., 2017). Based on M3 strain, we are considering constructing more strains with mutation in these proteins. Moreover, further studies are also needed to assess the safety and immunogenicity of these strains in larger animals such as rhesus macaques.

## Author Contributions

QL designed the experiments; XX, SF, XW, YH, XZ, and LW performed the experiments; XX, YZ, and YL performed the analyses; XX and MF wrote the manuscript; and all of the authors contributed to, read, and approved the final version of the manuscript.

## Conflict of Interest Statement

The authors declare that the research was conducted in the absence of any commercial or financial relationships that could be construed as a potential conflict of interest.
